# (*Z*)-4-(2-Hy­droxy­anilino)pent-3-en-2-one

**DOI:** 10.1107/S1600536812027894

**Published:** 2012-06-23

**Authors:** Benghanem Fatiha, Keraghel Saida, Chahmana Safia, Ourari Ali, Brelot Lydia

**Affiliations:** a Laboratoire d’Electrochimie, d’Ingenierie Moléculaire et de Catalyse Redox, Departement de Génie des Procédés, Faculté de Technologie, Université Ferhat Abbas, Sétif, Algeria; b Institut de Chimie de Strasbourg, UMR 7177 CNRS-UdS, Service de Radiocristallographie, 1 rue Blaise Pascal, 67008 Strasbourg Cedex, France

## Abstract

In the title compound, C_11_H_13_NO_2_, the dihedral angle between the planes defined by the 2-hy­droxy­phenyl­amino group and the pent-3-en-2-one mean plane [maximum deviations = 0.0275 (19) and 0.054 (2) Å, respectively] is 31.01 (10)°. There are intra­molecular bifurcated N—H⋯(O,O) hydrogen bonds involving the amine NH group and the adjacent carbonyl and hy­droxy O atoms. In the crystal, mol­ecules are linked *via* O—H⋯O hydrogen bonds, forming chains propagating along [100].

## Related literature

For transition metal complexes of Schiff bases, see: Salavati-Niasari (2006 ▶); Xiong *et al.* (2007 ▶); Basu *et al.* (2010 ▶). For the biological activity of Schiff bases, see: Jarrahpour *et al.* (2007 ▶); El-Masry *et al.* (2000 ▶); Singh & Dash (1988 ▶). For the use of Schiff bases as inter­midiates in many industrial processes, see: Salavati-Niasari & Nezamoddin Mirsattari (2007 ▶); Katsuki (1995 ▶); Ahamad *et al.* (2010 ▶); Da Silva *et al.* (2010 ▶); Soltani *et al.* (2010 ▶). For the tautomeric properties and conformations of the title compound, see: Kabak *et al.* (1998 ▶). For the photoconductivity of the title compound, see: Parekh *et al.* (2007 ▶), and for its thermochromic properties, see: Moustakali-Mavridis *et al.* (1978 ▶); Hadjoudis *et al.* (1987 ▶). For hydrogen bonding and graph-set notation, see: Bernstein *et al.* (1995 ▶).
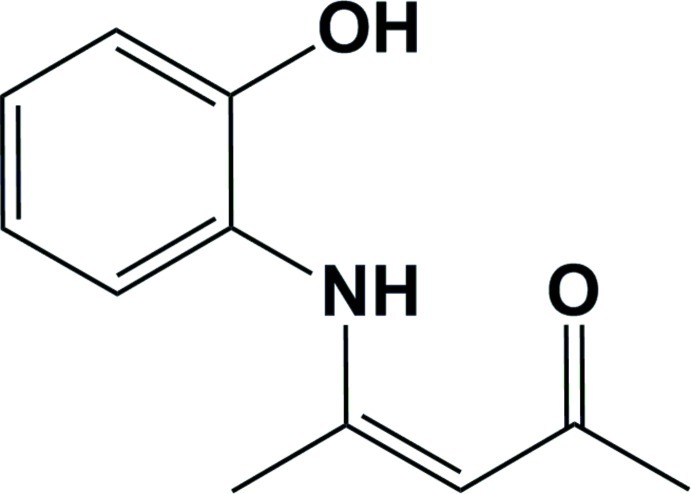


## Experimental

### 

#### Crystal data

C_11_H_13_NO_2_
*M* *_r_* = 191.22
Orthorhombic, 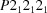

*a* = 8.7826 (4) Å
*b* = 10.3999 (5) Å
*c* = 11.1827 (3) Å
*V* = 1021.41 (7) Å^3^
*Z* = 4
Mo *K*α radiation
μ = 0.09 mm^−1^
*T* = 173 K
0.50 × 0.30 × 0.20 mm

#### Data collection

Nonius KappaCCD diffractometer
7005 measured reflections
1359 independent reflections
1260 reflections with *I* > 2σ(*I*)
*R*_int_ = 0.105

#### Refinement

*R*[*F* ^2^ > 2σ(*F* ^2^)] = 0.047
*wR*(*F* ^2^) = 0.126
*S* = 1.07
1359 reflections
137 parameters
H atoms treated by a mixture of independent and constrained refinement
Δρ_max_ = 0.22 e Å^−3^
Δρ_min_ = −0.29 e Å^−3^

### 

Data collection: *COLLECT* (Nonius, 1998 ▶); cell refinement: *DENZO* (Otwinowski & Minor, 1997 ▶); data reduction: *DENZO*; program(s) used to solve structure: *SHELXS97* (Sheldrick, 2008 ▶); program(s) used to refine structure: *SHELXL97* (Sheldrick, 2008 ▶); molecular graphics: *ORTEP-3 for Windows* (Farrugia, 1997 ▶) and *PLATON* (Spek, 2009 ▶); software used to prepare material for publication: *publCIF* (Westrip, 2010 ▶).

## Supplementary Material

Crystal structure: contains datablock(s) I, global. DOI: 10.1107/S1600536812027894/su2455sup1.cif

Structure factors: contains datablock(s) I. DOI: 10.1107/S1600536812027894/su2455Isup2.hkl

Supplementary material file. DOI: 10.1107/S1600536812027894/su2455Isup3.cml

Additional supplementary materials:  crystallographic information; 3D view; checkCIF report

## Figures and Tables

**Table 1:** Hydrogen-bond geometry (Å, °)

*D*—H⋯*A*	*D*—H	H⋯*A*	*D*⋯*A*	*D*—H⋯*A*
N1—H1*N*⋯O1	0.96 (3)	2.28 (3)	2.633 (2)	100.9 (18)
N1—H1*N*⋯O2	0.96 (3)	1.85 (3)	2.641 (2)	139 (2)
O1—H1⋯O2^i^	0.93 (3)	1.72 (3)	2.637 (2)	172 (3)

Symmetry code: (i) 
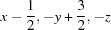
.

**Fig. 1.**
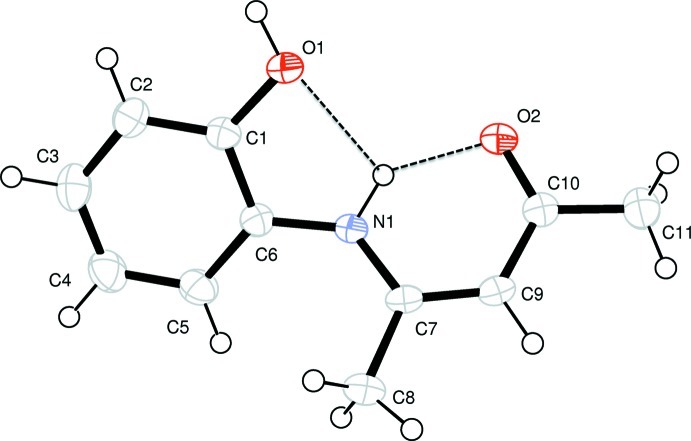
A view of the molecular structure of the title molecule, with the atom numbering. Displacement ellipsoids are drawn at the 50% probability level. The intramolecular bifurcated N-H···O/O hydrogen bond are shown as dashed lines (see Table 1 for details).

**Fig. 2.**
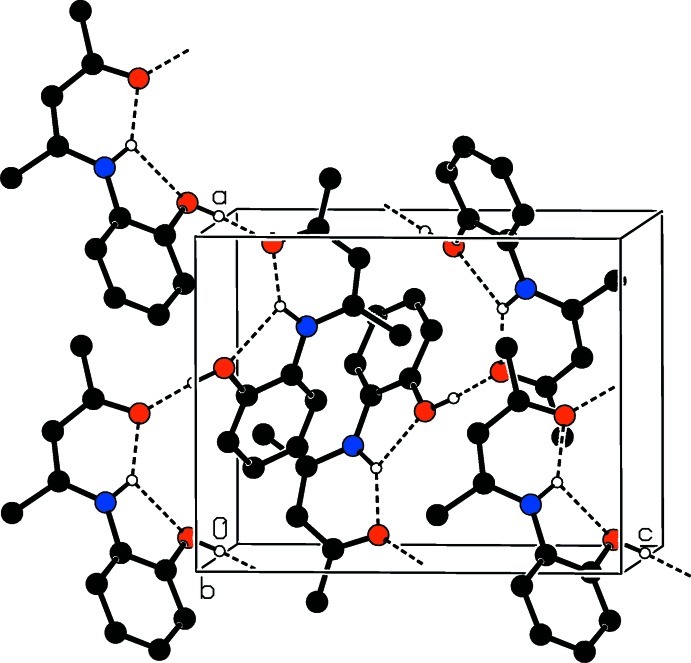
The crystal packing of the title compound viewed along the b axis. The N-H···O and O-H···O hydrogen bonds are shown as dashed lines (see Table 1 for details).

## supplementary crystallographic information

### Comment

Schiff bases have played an important role in the development of coordination chemistry. They have a great capacity for complexation of transition metal (Salavati-Niasari, 2006; Xiong *et al.*, 2007; Basu *et al.*, 2010). A literature survey revealed that this kind of compound possesses diverse biological activities such as antimicrobial (Jarrahpour *et al.*, 2007; El-Masry *et al.*, 2000) and antifungal (Singh & Dash, 1988). They also serve as intermediates in many industrial processes (Salavati-Niasari & Nezamoddin Mirsattari, 2007; Katsuki, 1995) and are used as corrosion inhibitors (Ahamad *et al.*, 2010; Da Silva *et al.*, 2010; Soltani *et al.*, 2010). In order to expand this field of research the title compound, a novel Schiff base derived from a non-aromatic aldehyde, has been synthesized and its crystal structure is reported herein.

The tautomeric properties and conformations of the title compound were investigated by (Kabak *et al.*, 1998). The enol tautomer form of the crystal of the title compound is established by the present crystal structure analysis. The positive photoconductivity of this compound has been studied by (Parekh *et al.*, 2007). The crystal exhibits positive photoconductivity and poor NLO responses, and it is photochromic not thermochromic in the solid state (Moustakali-Mavridis *et al.*, 1978; Hadjoudis *et al.*, 1987).

The molecular structure of the title molecule is shown in Fig. 1. It was prepared by the condensation of acetylacetone and 2-aminophenol and crystallized in the chiral space group P2_1_2_1_2_1_.

The molecule is not planar and the dihedral angle between the two planes defined by O1,C1-C6,N1 and O2,C7-C11 is equal to 31.01 (10) °. The small value of bond N1—C7 (1.346 (2) A°) in comparison to bond N1—C6 (1.416 (3) A°) results in a significant change in the bond angle C7—N1—C6 of 130.76 (17)°. The difference in the C—N bond distances is assumed to be due to the presence of the carbonyl group located at the C10 position. Shortening of the C7—N1 bond length and the large value of the C7—N1—C6 bond angle leads to the existence of an N1—H1···O2 intramolecular hydrogen bond (Table 1) with an S(6) ring motif (Bernstein *et al.*, 1995). The second intramolecular N1-H1···O1 hydrogen bond gives an S(5) ring motif.

In the crystal, molecules are linked via O-H···O hydrogen bonds to form chains propagating along [100] - see Table 1 and Fig. 2.

### Experimental

To a ethanol solution (5 ml) of (0.109 g, 1 mmol) of 2-aminophenol was slowly added a ethanol solution (5 ml) of acetylacetone (0.1 g, 1 mmol). The mixture was refluxed with constant stirring under a nitrogen atmosphere for 5 h. The mixture was removed and allowed to cool to rt and the solvent to evaporate slowly. After 20 days yellow crystals of the title compound were obtained. They were washed with ethanol then with diethyl ether.

### Refinement

The OH and NH H atoms were located in a difference Fourier map and freely refined. The C-bound H atoms were included in calculated positions and refined using a riding model: C—H = 0.95 and 0.98 Å for CH and CH_3_ H atoms, respectively, with *U*_iso_(H) = k × *U*_eq_(C) where k = 1.5 for methyl H atoms and = 1.2 for other H atoms. In the final cycles of refinement, in the absence of significant anomalous scattering effects, the Friedel pairs were merged and Δf " set to zero.

### Crystal data

**Table:**

C_11_H_13_NO_2_	*F*(000) = 408
*M**_r_* = 191.22	*D*_x_ = 1.244 Mg m^−^^3^
Orthorhombic, *P*2_1_2_1_2_1_	Mo *K*α radiation, λ = 0.71073 Å
Hall symbol: P 2ac 2ab	Cell parameters from 4219 reflections
*a* = 8.7826 (4) Å	θ = 1.0–27.5°
*b* = 10.3999 (5) Å	µ = 0.09 mm^−^^1^
*c* = 11.1827 (3) Å	*T* = 173 K
*V* = 1021.41 (7) Å^3^	Prism, yellow
*Z* = 4	0.50 × 0.30 × 0.20 mm

### Data collection

**Table:**

Nonius KappaCCD diffractometer	1260 reflections with *I* > 2σ(*I*)
Radiation source: fine-focus sealed tube	*R*_int_ = 0.105
Graphite monochromator	θ_max_ = 27.5°, θ_min_ = 2.7°
phi and ω scans	*h* = −10→11
7005 measured reflections	*k* = −12→13
1359 independent reflections	*l* = −14→12

### Refinement

**Table:**

Refinement on *F*^2^	Primary atom site location: structure-invariant direct methods
Least-squares matrix: full	Secondary atom site location: difference Fourier map
*R*[*F*^2^ > 2σ(*F*^2^)] = 0.047	Hydrogen site location: inferred from neighbouring sites
*wR*(*F*^2^) = 0.126	H atoms treated by a mixture of independent and constrained refinement
*S* = 1.07	*w* = 1/[σ^2^(*F*_o_^2^) + (0.0792*P*)^2^ + 0.0946*P*] where *P* = (*F*_o_^2^ + 2*F*_c_^2^)/3
1359 reflections	(Δ/σ)_max_ < 0.001
137 parameters	Δρ_max_ = 0.22 e Å^−^^3^
0 restraints	Δρ_min_ = −0.29 e Å^−^^3^

### Special details

**Table:**

Geometry. Bond distances, angles etc. have been calculated using the rounded fractional coordinates. All su's are estimated from the variances of the (full) variance-covariance matrix. The cell esds are taken into account in the estimation of distances, angles and torsion angles
Refinement. Refinement of *F*^2^ against ALL reflections. The weighted *R*-factor *wR* and goodness of fit *S* are based on *F*^2^, conventional *R*-factors *R* are based on *F*, with *F* set to zero for negative *F*^2^. The threshold expression of *F*^2^ > σ(*F*^2^) is used only for calculating *R*-factors(gt) *etc*. and is not relevant to the choice of reflections for refinement. *R*-factors based on *F*^2^ are statistically about twice as large as those based on *F*, and *R*- factors based on ALL data will be even larger.

### Fractional atomic coordinates and isotropic or equivalent isotropic displacement parameters (Å^2^)

**Table:**

	*x*	*y*	*z*	*U*_iso_*/*U*_eq_	
O1	0.58388 (17)	0.70724 (15)	0.03852 (14)	0.0320 (4)	
O2	0.95582 (17)	0.68677 (15)	0.15060 (12)	0.0331 (4)	
N1	0.69854 (19)	0.57884 (17)	0.22040 (14)	0.0248 (4)	
C1	0.4942 (2)	0.61181 (19)	0.08258 (17)	0.0257 (5)	
C2	0.3527 (2)	0.5805 (2)	0.03503 (19)	0.0322 (6)	
C3	0.2679 (3)	0.4816 (2)	0.0850 (2)	0.0387 (7)	
C4	0.3242 (3)	0.4126 (2)	0.1812 (2)	0.0402 (7)	
C5	0.4665 (3)	0.4415 (2)	0.2282 (2)	0.0349 (6)	
C6	0.5510 (2)	0.54332 (18)	0.18138 (17)	0.0256 (5)	
C7	0.7611 (2)	0.57778 (19)	0.33025 (16)	0.0253 (5)	
C8	0.6698 (3)	0.5315 (3)	0.43570 (18)	0.0392 (7)	
C9	0.9069 (2)	0.6227 (2)	0.34810 (17)	0.0276 (5)	
C10	0.9993 (2)	0.6778 (2)	0.25800 (17)	0.0275 (5)	
C11	1.1530 (3)	0.7300 (3)	0.2911 (2)	0.0400 (7)	
H1	0.542 (3)	0.738 (3)	−0.032 (3)	0.044 (7)*	
H1N	0.762 (3)	0.619 (3)	0.162 (2)	0.033 (6)*	
H2	0.31400	0.62690	−0.03160	0.0390*	
H3	0.17070	0.46110	0.05310	0.0460*	
H4	0.26530	0.34520	0.21510	0.0480*	
H5	0.50630	0.39190	0.29240	0.0420*	
H8A	0.56570	0.56470	0.42990	0.0590*	
H8B	0.71680	0.56240	0.50980	0.0590*	
H8C	0.66760	0.43730	0.43600	0.0590*	
H9	0.94840	0.61620	0.42630	0.0330*	
H11A	1.23200	0.68290	0.24730	0.0600*	
H11B	1.16920	0.71960	0.37730	0.0600*	
H11C	1.15810	0.82140	0.27030	0.0600*	

### Atomic displacement parameters (Å^2^)

**Table:**

	*U*^11^	*U*^22^	*U*^33^	*U*^12^	*U*^13^	*U*^23^
O1	0.0344 (8)	0.0336 (8)	0.0281 (7)	−0.0051 (7)	−0.0054 (6)	0.0101 (6)
O2	0.0357 (7)	0.0395 (8)	0.0241 (7)	−0.0059 (7)	0.0004 (6)	0.0062 (6)
N1	0.0271 (8)	0.0257 (8)	0.0217 (7)	−0.0021 (7)	0.0009 (6)	0.0040 (6)
C1	0.0274 (9)	0.0234 (9)	0.0263 (9)	0.0023 (8)	0.0021 (8)	0.0009 (7)
C2	0.0305 (10)	0.0324 (10)	0.0336 (10)	0.0031 (10)	−0.0033 (8)	−0.0031 (9)
C3	0.0308 (10)	0.0342 (11)	0.0511 (13)	−0.0023 (10)	−0.0051 (10)	−0.0075 (10)
C4	0.0403 (12)	0.0300 (11)	0.0503 (14)	−0.0086 (11)	−0.0004 (10)	0.0027 (10)
C5	0.0407 (12)	0.0263 (10)	0.0377 (11)	−0.0044 (10)	−0.0006 (9)	0.0057 (8)
C6	0.0271 (9)	0.0236 (9)	0.0261 (9)	0.0004 (8)	−0.0002 (7)	−0.0003 (7)
C7	0.0319 (9)	0.0236 (8)	0.0205 (9)	0.0043 (8)	0.0006 (7)	0.0043 (7)
C8	0.0406 (12)	0.0513 (13)	0.0258 (10)	−0.0059 (12)	0.0040 (9)	0.0093 (10)
C9	0.0324 (10)	0.0281 (10)	0.0223 (8)	0.0029 (9)	−0.0028 (8)	0.0042 (7)
C10	0.0304 (9)	0.0250 (9)	0.0272 (9)	0.0023 (9)	0.0004 (7)	−0.0007 (7)
C11	0.0374 (11)	0.0434 (13)	0.0393 (11)	−0.0088 (12)	−0.0011 (10)	−0.0005 (10)

### Geometric parameters (Å, º)

**Table:**

O1—C1	1.359 (2)	C9—C10	1.415 (3)
O2—C10	1.264 (2)	C10—C11	1.501 (3)
O1—H1	0.93 (3)	C2—H2	0.9500
N1—C6	1.416 (2)	C3—H3	0.9500
N1—C7	1.346 (2)	C4—H4	0.9500
N1—H1N	0.96 (3)	C5—H5	0.9500
C1—C2	1.390 (3)	C8—H8A	0.9800
C1—C6	1.406 (3)	C8—H8B	0.9800
C2—C3	1.387 (3)	C8—H8C	0.9800
C3—C4	1.385 (3)	C9—H9	0.9500
C4—C5	1.389 (4)	C11—H11A	0.9800
C5—C6	1.395 (3)	C11—H11B	0.9800
C7—C8	1.505 (3)	C11—H11C	0.9800
C7—C9	1.378 (3)		
			
C1—O1—H1	109.3 (18)	C3—C2—H2	120.00
C6—N1—C7	130.74 (16)	C2—C3—H3	120.00
C6—N1—H1N	115.9 (15)	C4—C3—H3	120.00
C7—N1—H1N	112.9 (15)	C3—C4—H4	120.00
O1—C1—C2	123.38 (18)	C5—C4—H4	120.00
O1—C1—C6	116.69 (16)	C4—C5—H5	120.00
C2—C1—C6	119.93 (18)	C6—C5—H5	120.00
C1—C2—C3	120.0 (2)	C7—C8—H8A	109.00
C2—C3—C4	120.4 (2)	C7—C8—H8B	109.00
C3—C4—C5	120.2 (2)	C7—C8—H8C	109.00
C4—C5—C6	120.1 (2)	H8A—C8—H8B	109.00
N1—C6—C1	115.76 (16)	H8A—C8—H8C	110.00
N1—C6—C5	124.68 (18)	H8B—C8—H8C	109.00
C1—C6—C5	119.39 (18)	C7—C9—H9	118.00
C8—C7—C9	119.35 (17)	C10—C9—H9	118.00
N1—C7—C8	120.02 (17)	C10—C11—H11A	109.00
N1—C7—C9	120.60 (17)	C10—C11—H11B	109.00
C7—C9—C10	124.56 (17)	C10—C11—H11C	109.00
O2—C10—C9	122.23 (17)	H11A—C11—H11B	109.00
O2—C10—C11	118.64 (18)	H11A—C11—H11C	110.00
C9—C10—C11	119.12 (17)	H11B—C11—H11C	110.00
C1—C2—H2	120.00		
			
C6—N1—C7—C9	−177.06 (19)	C1—C2—C3—C4	−0.8 (3)
C7—N1—C6—C1	148.6 (2)	C2—C3—C4—C5	−0.3 (3)
C7—N1—C6—C5	−36.2 (3)	C3—C4—C5—C6	2.3 (3)
C6—N1—C7—C8	0.8 (3)	C4—C5—C6—N1	−178.26 (19)
C6—C1—C2—C3	−0.1 (3)	C4—C5—C6—C1	−3.2 (3)
O1—C1—C6—N1	−2.5 (3)	N1—C7—C9—C10	3.0 (3)
O1—C1—C6—C5	−177.92 (18)	C8—C7—C9—C10	−174.9 (2)
O1—C1—C2—C3	179.93 (18)	C7—C9—C10—O2	−2.2 (3)
C2—C1—C6—N1	177.60 (17)	C7—C9—C10—C11	176.5 (2)
C2—C1—C6—C5	2.1 (3)		

### Hydrogen-bond geometry (Å, º)

**Table:**

*D*—H···*A*	*D*—H	H···*A*	*D*···*A*	*D*—H···*A*
N1—H1*N*···O1	0.96 (3)	2.28 (3)	2.633 (2)	100.9 (18)
N1—H1*N*···O2	0.96 (3)	1.85 (3)	2.641 (2)	139 (2)
O1—H1···O2^i^	0.93 (3)	1.72 (3)	2.637 (2)	172 (3)

Symmetry code: (i) *x*−1/2, −*y*+3/2, −*z*.
